# Medicolegal and Ethical Challenges in Diagnosing and Managing Factitious Disorder Imposed on Another (FDIA): A Case Report

**DOI:** 10.7759/cureus.31513

**Published:** 2022-11-14

**Authors:** Olumuyiwa Fatade, Omotola K Ajibade

**Affiliations:** 1 Department of Psychiatry and Behavioral Sciences, Hackensack Meridian Ocean University Medical Center, Brick, USA

**Keywords:** caregivers, medicolegal, diabetes, patient safety, guardianship, munchausen syndrome by proxy, munchausen syndrome, factitious disorder by proxy, factitious disorder, fdia

## Abstract

Factitious disorder (FD) is a condition in which patients fabricate evidence and produce false stories that often subject them to needless medical interventions with no clear benefits. In some instances, it can be imposed on a secondary victim often as a form of abuse. Most often, victims of a factitious disorder imposed on another (FDIA) are children or the elderly. Despite a mortality rate between 6 and 10% among victims, FDIA still remains underdiagnosed. Research on it often fails to address healthcare management initiatives, as well as the legal and ethical challenges physicians must navigate when managing it. In this report, we present a rare case of FDIA in an adult patient with a history of diabetes, substance use disorder, and schizoaffective disorder.

This case highlights the importance of appropriate communication and detailed documentation when signs of FDIA are suspected. It also identifies the benefits of implementing a multidisciplinary approach when appropriate to minimize harm and improve outcomes.

## Introduction

Factitious disorder (FD) has been recognized as a serious medical condition for over a century. It was initially labeled as Munchausen syndrome by British psychiatrist Richard Asher, who used the term to describe a chronic subtype of FD where patients consistently fabricated evidence and produced false stories that subjected them to needless medical investigations and treatments [1]. Later, the term Munchausen syndrome by proxy (MSbP) was coined by English pediatrician Roy Meadow, to describe a form of abuse whereby parents fabricated illnesses in their children [2]. In this form of the disorder, caregivers falsify medical or psychological illnesses in others without any apparent external reward. This deceptive behavior can cause others to have an overestimated view of the victim’s illness or level of impairment, often leading to unnecessary or excessive medical interventions [3]. In the Diagnostic and Statistical Manual of Mental Disorders, Fifth Edition (DSM-5), MSbP is referred to as a factitious disorder imposed on another (FDIA), and its diagnosis requires that it not be better explained by other forms of mental illnesses.

Although some authors have focused on particular forms of FDIA to understand its incidence better, its prevalence in the United States (US) remains largely unknown. Ayoub et al. estimated that about 600 cases of suffocation and non-accidental poisoning linked to FDIA occur in the US every year [4]. Currently, the mortality rate among FDIA victims ranges between 6 and 10% [5]. However, these estimates do not account for the aspect of the perpetrator's deceit, comorbid illnesses, and the legal and ethical challenges involved in making an FDIA diagnosis. Research on the management of FDIA remains scarce; while many authors have focused on the peculiar features of both perpetrators and victims, which could aid in the diagnosis, they often fail to address the healthcare management initiatives and the legal and ethical challenges physicians must navigate when managing FDIA victims.

To tackle this challenge, we present a rare case of FDIA in an adult patient. The presentation and accurate assessment of the social dynamics and initiatives taken in this case can help clinicians avoid significant burdens to healthcare systems and, more importantly, improve the quality of care for these patients.

## Case presentation

The victim 

G, a 42-year-old man with a history of diabetes, hypercholesterolemia, hypertension, substance use disorder, stroke, and schizoaffective disorder was brought to the emergency department (ED) by his mother following multiple episodes of uncontrolled elevated blood glucose. Psychiatry was consulted due to G’s previous history of attempted suicide. G’s mother provided most of the details and history of her son's medical conditions. She reported that he had dropped out of school due to marijuana use in the 10th grade, after which he had lived with her for a prolonged period. During this time, he continued his marijuana use, started cocaine use, and received multiple legal charges for drug possession, panhandling, and shoplifting. He was never married, had no children, and remained unemployed. G’s mother reported that two years before his presentation, she had kicked him out of her home due to his continued substance use. Following this, he lived with his siblings and remained fiercely independent, often being diligent about his blood glucose management and checking himself into mental health facilities when experiencing suicidal ideations.

The perpetrator’s role and account 

G’s mother appeared disheveled, wearing multiple pieces of clothing, and carrying multiple bags that she guarded very closely. She was observed to be closely monitoring the nursing staff outside G’s room to see their pattern of movement and paid specific attention to shift changes. She reported that three months before G’s presentation to the ED, he had returned to live with her. He had been subsequently hospitalized for over 50 days following an “incidental insulin overdose”. At the time, he had developed a stroke which affected his speech patterns significantly and rendered him unable to care for himself. He had later been discharged to a rehab facility. She also noted bringing him to our facility three days before his current presentation and reported taking him back to the rehab facility following his stabilization as recommended.

Assessment

The psychiatric treatment team examined G with his mother at his side. He appeared younger than his stated age, disheveled, with unkempt hair, and of a medium build. He was in mild distress, diaphoretic and confused. His eye contact was evasive. He presented himself as someone anxious and seeking help. His attitude was one of submissiveness, often making multiple glances at his mother to seek her approval before answering any questions asked. He showed no unusual movements or gestures, and his speech was initially nonverbal. Later, he answered with significantly delayed latency of response, and a soft and slow speech rate with his mother interjecting to answer all questions before he could respond. He characterized his mood as “depressed” and his affect was flat. Thought content and process were difficult to be evaluated due to his mother’s constant interjections. He denied any current suicidal or homicidal ideations, as well as any current visual or auditory hallucinations. His decision-making capacity was limited, and further attempts to formally test the patient's cognitive status were impeded by the mother. Other routine blood tests were within normal limits and the urine drug screen was negative for any illicit substances. His point-of-care-test (POCT) blood glucose was found to be 196 mg/dL; he was stabilized, and hourly blood sugar checks were instituted.

Diagnosis of FDIA and outcome

Following the initial assessment of the patient, he had multiple hypoglycemic episodes while his mother was at his bedside. Subsequent follow-up evaluations were refused by his mother, including blood work and any further diagnostic testing. Furthermore, the treatment team identified numerous inconsistencies between the mother's account of the patient’s hospitalization and previous clinical documentation, which aided in the diagnosis of FDIA. Firstly, previous documentation noted that G’s “incidental insulin overdose” as reported by his mother was later determined to be another suicide attempt when he was questioned without his mother. During his 50-day hospitalization for insulin overdose, his mother was noted to be very disruptive to the treatment team and showed significant descriptive knowledge of his condition while refusing multiple treatments that would have accelerated his recovery. Similar observations were made at the nursing home where G was discharged during his stroke recovery. There, her disruptive behavior continued, and the nursing staff documented that G had multiple hypoglycemic episodes that only occurred when his mother was at his bedside. Her behavior prompted them to alert adult protective services (APS), but G’s mother had signed him out against medical advice (AMA) before the case could be evaluated. APS was also unable to reach them at the listed address, due to his mother bringing him to our hospital for evaluation. FD imposed on self, mismanaged diabetes, and somatic symptom disorders were initially considered, but G’s lack of hypoglycemic episodes in the absence of his mother, along with increased disingenuousness of her actions relating to his illness made FDIA more likely.

Given this information, the psychiatry treatment team approached the mother with a supportive confrontation that emphasized patient well-being and assurance that care would continue. She admitted not being truthful with the treatment team and noted not taking him to the rehab center following his discharge from our hospital three days prior. She also noted that she had signed him out against medical advice at previous clinical centers. Despite this, she remained disruptive in G’s management, refusing to allow follow-up interviews and treatments that would have stabilized his blood glucose.

The treatment team held a meeting with the ethics committee, all coordinating medical teams, social workers, concerned hospital administration personnel, in-house legal departments, nurses, and floor staff to coordinate G’s care. A decision was made to restrict his mother’s access to allow for a follow-up interview, stabilization of his blood glucose, and evaluation by APS. Since APS had been previously consulted at his previous rehab center, recommendations were made to apprise them of the new developments. Due to G’s inability to care for himself, and the increased risk of harm posed by his mother's refusal to allow life-saving treatments for him, the treatment team was advised of the need for temporary emergency guardianship.

Following the restrictions imposed on his mother, G’s blood glucose stabilized with no new occurrences of hypoglycemic episodes. In the following days, his mother was noted by the floor staff to take advantage of the shift changes to come back to the patient’s bedside unnoticed. She again resumed her refusal of her son's evaluation and treatment. Another meeting was held to coordinate and clarify shift change methodology and allow incoming staff to stay apprised of the situation. Stricter security measures were instituted. Consideration for the appointment of a Power of Attorney (POA) was made. However, due to the limitations in the patient’s capacity and the gravity of the situation, emergency guardianship was sought instead. Following the APS evaluation, emergency guardianship was approved, and G was stabilized and transferred to the nursing home.

## Discussion

Diagnostic challenges and healthcare initiatives 

This case highlights the importance of appropriate communication and detailed documentation when signs of FDIA are suspected. Early recognition can also greatly aid in reducing harm. Without appropriate documentation from previous caregivers, the treatment team would have struggled to identify the cause of G’s hypoglycemic episodes. Providers should be aware of warning signs that are common to FD, which generally include the involvement of multiple medical providers, symptoms that dramatically worsen or shift when the caregiver is present, and the presence of recurrent hospitalizations [6]. Many of these were present in G’s case and were often tied to the presence of his mother at the bedside. Clinicians should maintain a high level of suspicion when these signs are present, while also focusing on coordinating care between all involved providers. A multidisciplinary approach must be implemented to minimize victim harm because improved outcomes have been reported when elements of medical and psychological support are incorporated into a multidisciplinary team [7]. In this case, social workers and nurses were instrumental in identifying early warning signs that might have been overlooked. Additionally, G’s mother’s ability to take advantage of shift changes highlighted the importance of effective communication between non-psychiatrist caregivers as well. The transfer of essential patient information and responsibility for care between different healthcare providers is an integral part of communication in healthcare. Therefore, non-psychiatrists are usually the first to raise concerns regarding the presence of FDIA. When shift changes are inadequate, safety quickly becomes a concern; to avoid this, providers should work to promote appropriate hand-offs between caregivers at all levels [7,8]. When this was implemented in our case, G’s hypoglycemic episodes stopped and he was able to be transferred to a rehab center. 

Ethical and legal challenges

An FDIA diagnosis not supported by robust documentation could pose legal risks for providers. Unsupported allegations can increase the risk of liability for slanderous or defamatory statements. Furthermore, FDIA in an adult patient could be difficult to identify and pose different legal and ethical challenges than in pediatric cases. Unlike in pediatric patients where the majority of caregivers are parents, perpetrators of adult FDIA are more diverse and can include spouses and children [9]. Special attention should be paid to the patient's decisional capacity and competency in case legal and ethical questions arise. While our case occurred in New Jersey, each state might have different methods of securing emergency guardianship. One factor that greatly aided our decision-making involved elucidating whether the caregiver was also the patient’s POA or the legal guardian. In some situations, appointing a POA may be more appropriate than seeking guardianship. This is particularly salient if the patient is deemed to have the clinical capacity or the legal competence to make such decisions. We recommend making this determination as soon as possible when FDIA is suspected. In our case, this caregiver was not the POA and it was not clear that the patient could appoint one on his behalf. Caregivers diagnosed with FDIA could litigate and challenge the diagnosis when guardianship disputes arise. Due to the nature of the diagnosis characterizing the caregiver's behavior, a wrong diagnosis could strain relationships between caregivers and victims. The risk of an incorrect diagnosis is often at odds with federally mandated reporting standards outlined in such legislation as the Elder Justice Act of 2009. Physicians should seek appropriate legal advice from both the hospital ethics committee and in-house legal departments when considering emergency guardianship.

Adult victims of FDIA should also receive the same level of protection, rights to privacy, and confidentiality provided to any other patient. Despite G’s disheveled appearance and his mother’s suspicious bags, searching them without appropriate consent would have violated their privacy. Similarly, notifying other hospitals or circulating a blacklist of such caregivers and patients should be highly discouraged [10]. This is because it could violate physicians' ethical and legal responsibilities. Even though FDIA could lead to wasteful use of resources, physicians should make their own independent assessments and diagnoses based on appropriate documentation and patient and collateral histories. To identify inappropriate caregiver behaviors without violating legal or ethical concerns, some have suggested the use of video cameras. If covert video cameras are already in routine use, and hospitals post visible signs informing patients and visitors of their use, legal privacy concerns could be satisfied. Video surveillance of caregivers suspected of FDIA is highly effective in identifying inappropriate behavior [10]. Perhaps the most important aspect of managing FDIA is the immediate steps that should be taken to reduce patient harm once the condition is identified. Providers must remain vigilant during their assessment to avoid overlooking harmful signs.

## Conclusions

FDIA is an underdiagnosed form of abuse that requires a high index of suspicion and detailed documentation to diagnose. Effective communication between all providers can help physicians navigate the legal and ethical challenges that can arise when making an FDIA diagnosis. This case required the use of emergency guardianship to reduce patient harm. While this might not be necessary in all cases of FDIA, it is important to clearly understand the laws, policies, and procedures applicable when such cases are suspected. Physicians should be aware of the victim’s right to privacy and should apply the same level of care and protection that they would apply to any other patient. When implemented appropriately, multidisciplinary healthcare initiatives could lead to positive outcomes.
